# Multidisciplinary adolescent and young adult neuro-oncology clinic: Clinical cases, practice challenges, and future perspectives

**DOI:** 10.1093/noajnl/vdaf072

**Published:** 2025-04-11

**Authors:** Christianne V Mojica, Thiago Pimentel Muniz, Xin Wang, Stephanie Baker, Kim Edelstein, Cheryl Kanter, Katherine Mileski, Candice Nguyen, Angela Sekely, Derek S Tsang, Cynthia Hawkins, Uri Tabori, Warren P Mason, Julie Bennett

**Affiliations:** Department of Medicine, Division of Neurology and Medical Oncology and Hematology, Princess Margaret Cancer Centre, Toronto, Ontario, Canada; Department of Medicine, Division Medical Oncology and Hematology, Princess Margaret Cancer Centre, Toronto, Ontario, Canada; Department of Medicine, Division Medical Oncology and Hematology, Princess Margaret Cancer Centre, Toronto, Ontario, Canada; Cancer Clinical Research Unit, Princess Margaret Cancer Centre, Toronto, Ontario, Canada; Department of Supportive Care, Princess Margaret Cancer Centre, Toronto, Ontario, Canada; Department of Supportive Care, Princess Margaret Cancer Centre, Toronto, Ontario, Canada; Pencer Brain Tumor Centre, Princess Margaret Cancer Centre, Toronto, Ontario, Canada; Clinical Nutrition Department, Princess Margaret Cancer Centre, Toronto, Ontario, Canada; Department of Supportive Care, Princess Margaret Cancer Centre, Toronto, Ontario, Canada; Radiation Medicine Program, Princess Margaret Cancer Centre, Toronto, Ontario, Canada; Division of Hematology/Oncology, The Hospital for Sick Children, Toronto, Ontario, Canada; Division of Hematology/Oncology, The Hospital for Sick Children, Toronto, Ontario, Canada; Department of Medicine, Division of Neurology and Medical Oncology and Hematology, Princess Margaret Cancer Centre, Toronto, Ontario, Canada; Division of Hematology/Oncology, The Hospital for Sick Children, Toronto, Ontario, Canada; Department of Medicine, Division Medical Oncology and Hematology, Princess Margaret Cancer Centre, Toronto, Ontario, Canada

**Keywords:** AYA, multidisciplinary care, neuro-oncology

## Abstract

**Background:**

The distinct tumor histopathology, molecular features, and psychosocial needs among adolescents and young adults (AYA) with brain tumors pose challenges within traditional healthcare systems. Establishing a multidisciplinary AYA neuro-oncology clinic has been proposed to address these gaps in care. This is the first study to describe the framework and patient profile of a multidisciplinary AYA neuro-oncology clinic in a quaternary cancer center in Canada.

**Methods:**

Clinic framework was outlined and patients seen from December 2022 to June 2024 were included. Demographic profiles, tumor characteristics, treatment details, clinical trial enrollment, and allied health referrals were collected. Barriers encountered were summarized.

**Results:**

The clinic is composed of specialists in pediatric and adult neuro-oncology with seamless referrals to neurosurgery, radiation oncology, and allied health teams. A total of 100 patients (males 54%, females 46%) were seen with a median age of 24 years. Pediatric-type low-grade glioma (PLGG) was the leading diagnosis. BRAF alterations were the primary molecular drivers. Twenty-nine patients received active neuro-oncology management in the clinic. Overall, 77 patients underwent at least one surgery, 31 patients received radiotherapy, and 43 patients received chemotherapy. Trametinib was the primary targeted treatment prescribed. Three patients were eligible and enrolled in clinical trials. Barriers identified included a lack of peer support groups and a paucity of available clinical trials.

**Conclusions:**

This study provides insight into the clinical profile of patients seen in a multidisciplinary AYA neuro-oncology clinic in Canada. Multidisciplinary care is feasible and integral in addressing the multifaceted needs of AYAs with brain tumors.

**Key Points**

- A multidisciplinary approach is necessary to address the distinct needs and characteristics of adolescents and young adults (AYA) patients.
- Establishing a multidisciplinary AYA neuro-oncology clinic with pediatric and adult specialists and allied health teams is feasible.
- This clinic framework serves as a model for providing integrated care to AYA patients.

**Importance of the Study**

Multidisciplinary adolescents and young adults (AYA) neuro-oncology clinics have been recommended to address the fragmented care of this unique subgroup of patients; however, there have been no published data on the framework and conduct of such a clinical model. This is the first review to describe the experiences of a multidisciplinary AYA neuro-oncology clinic in a quaternary cancer center in Canada. The clinical profile of patients provides insight into this population as well as the treatment practices in this specific setting. Barriers, areas for improvement, and future directions are outlined. Ultimately, this study highlights the feasibility of a multidisciplinary clinic in providing holistic and personalized care to AYA neuro-oncology patients. This multidisciplinary approach should be used in the care of AYA patients with brain tumors in other geographic settings.

There has been growing interest in the care of adolescent and young adult (AYA) patients with cancer, typically defined as patients aged 15–39 years.^1^ In the United States, the incidence rate of cancer is 77.9 per 100 000 AYAs.^2^ In 2024, an estimated 84 100 AYAs would be diagnosed with new cancers^3^ and 13 350 AYAs would be diagnosed with new brain tumors in the United States.^4^ Cancer-related mortality rate in this age group has not significantly decreased over time.^2,4^ The 2024 report from the Central Brain Tumor Registry of the United States (CBTRUS) documents that primary central nervous system (CNS) tumors account for the leading cause of cancer-related death among AYAs in the 15–24 years age group and the second leading cause of death among all AYAs with cancer.^4^ Furthermore, CNS malignancy ranked second among cancers with the most disability-adjusted life-years, second to breast cancer in AYAs.^5^ Despite statistics that highlight the significant global burden of CNS cancer among the AYA population, the delivery of care to these patients has been limited by the unique characteristics and needs of this age group which are not readily addressed by traditional healthcare systems.^1,6,7^

The molecular and histopathologic characteristics of brain tumors differ in the AYA age group compared with the pediatric and older adult populations and most of these features are still poorly understood.^4,6,8^ The 2021 WHO CNS classification subdivided glioma into pediatric-type (including diffuse low-grade glioma, MAPK pathway-altered and diffuse midline glioma, H3 K27-altered) and adult-type (including IDH-mutant glioma and IDH-wild-type glioblastoma),^9^ both of which are prevalent in AYA neuro-oncology patients.^4^ Adult medical practitioners have less experience with pediatric-type tumors that are also common in AYA. There is little study of biology in this age group and treatment often varies from typical therapies offered to adult patients, including targeted therapy and intensive chemotherapy.

Higher enrollment rates in clinical trials affect treatment practices and subsequently, survival rates.^10^ A study found a correlation between the clinical trial enrollment rate and the trend in the decrease in cancer-related mortality among young adults in the United States.^11^ The low clinical trial participation among AYAs is thought to contribute to the lack of improvement in the survival trend in this age group.^10–12^ A review from Chicago showed that while 38% of neuro-oncology AYA patients seen in a children’s hospital participated in a clinical trial, only 12.6% of AYA patients seen in the affiliated adult cancer center participated.^13^ A recent study looking at clinical trial engagement among AYA neuro-oncology patients found several challenges including lack of physician expertise, specific patient characteristics such as preferences and psychosocial background, varying tumor features, limitations to access, and overall paucity of available trials.^14^ AYAs also face unique psychosocial challenges which further add to the complexity of care such as navigating between the disease and the shifts in their self-image, careers, education, and social relationships, in the background of the financial burden that a cancer diagnosis entails.^1,15^

In Canada, there have been several initiatives to address the gaps in the care of AYA with brain tumors. The Canadian AYA Neuro-oncology Network (CANON), a group comprising pediatric and adult neuro-oncologists, neuropathologists, neuroradiologists, and neurosurgeons with a special interest in the diagnosis and care of AYAs with CNS tumors was formed.^16^ Since 2021, this group has conducted biweekly meetings to discuss challenging cases and review new literature on the diagnosis and treatment of tumors.^17^ This group has also developed Canadian guidelines on molecular testing of CNS tumors in AYAs.^18^ Despite these pioneering works, there is still a paucity of published literature on the care of AYA neuro-oncology patients.^6,13^ The formation of multidisciplinary AYA neuro-oncology programs in major cancer hospitals has been recommended to bridge this gap in care.^6,7,19^

A dedicated AYA neuro-oncology clinic at Princess Margaret Cancer Center was established in December 2022 based on an appreciation of the distinct molecular characteristics of AYA CNS cancer and the recognition of the unique treatments administered to this patient population.^4,6,8^ It is important to highlight that referrals to the AYA clinic were based on tumor biology rather than patient age. This clinic is led by a pediatric neuro-oncologist, with the aim to care for AYA patients with CNS tumors typically found in the pediatric age group, while patients with adult-type tumors remain in the care of the well-established adult neuro-oncology practice. The scope of the AYA clinic includes glioma with RAS/MAPK alterations, embryonal tumors, CNS germ cell tumors, craniopharyngioma, CNS tumors with germline cancer predisposition syndromes (including NF-1, tuberous sclerosis complex, MMRD, DICER1-predisposition) and patients transitioning to active medical care at an adult cancer center from the pediatric neuro-oncology program. AYA patients with adult-type tumors (such as IDH-mutant glioma, IDH wild-type glioblastoma, meningioma, and schwannoma) typically remained in the care of the well-established adult neuro-oncology practice. Notably, the AYA clinic is distinct from the survivorship clinic, where neuro-oncology patients are referred for ongoing aftercare follow-up.

This is the first study to describe the conduct of a multidisciplinary AYA neuro-oncology clinic in Canada and the clinical profile of the patients including a comprehensive review of their tumor markers, radiographic features, treatment regimens, clinical trial enrollment rate, and referral to other services. The goal of the study is to describe the multidisciplinary framework of the AYA neuro-oncology clinic, provide insight into the clinical profile of the patients and the treatment practices in this setting, describe the challenges to conducting care, and formulate recommendations for the clinic.

## Methodology

This is a retrospective review of the clinical profile of all patients seen in the AYA neuro-oncology clinic at the Princess Margaret Cancer Center. All new patients and referrals evaluated in the clinic from December 2022 to June 2024 were included. Cases with informal consults through e-mails or discussions during virtual rounds with other hospitals were excluded. After identifying the patients, data were collected through chart review in the hospital’s electronic medical records. Data collected include the following variables: age, sex, clinical diagnosis, cancer predisposition, pathology findings (ie, molecular features), radiographic features (ie, primary site of disease, metastasis if present), treatment details (type of surgery, radiotherapy, chemotherapy, targeted treatment, and/or other treatment modalities), clinical trial enrollment, and referrals to specialty clinics and allied health teams such as neuropsychology, psychosocial oncology, clinical dietician, fertility clinic, and rehabilitation or physical therapy. Quantitative data were summarized using descriptive statistics. The framework of the multidisciplinary clinic is described and the challenges encountered are summarized.

## Results

### Multidisciplinary AYA Neuro-oncology Clinic Framework

**Figure 1.**
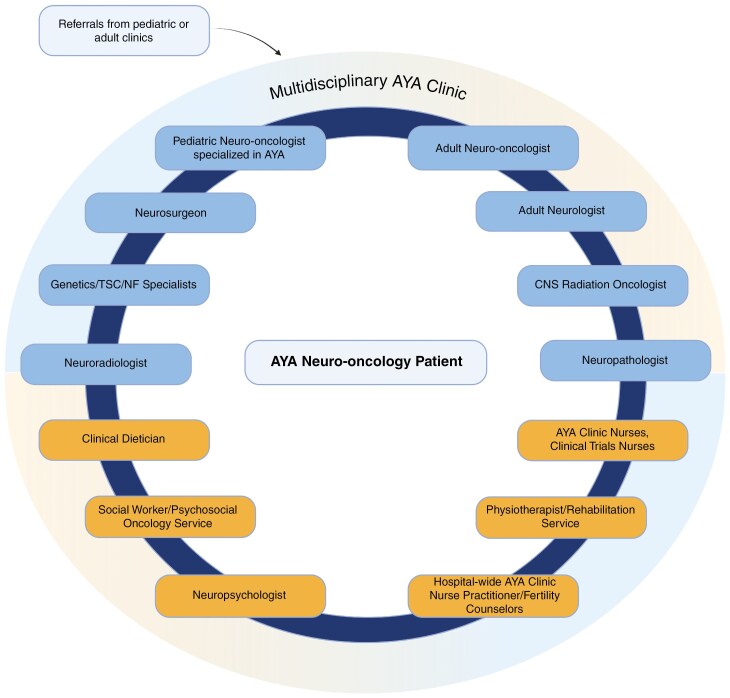
Multidisciplinary adolescents and young adults Neuro-oncology Clinic Framework. Patients are referred from pediatric and adult clinics. The multidisciplinary clinic is composed of the medical and allied health teams. TSC, Tuberous sclerosis complex; NF, Neurofibromatosis. (Created in BioRender. Mojica, C. (2025) https://BioRender.com/g07d240.

The clinic is led by a pediatric neuro-oncologist with specialized training in the AYA population and an adult medical oncologist with training in CNS tumors. Referrals come from various sources. Patients with pediatric-type tumors from the affiliate pediatric center are transitioned to the AYA clinic once they turn 18 years old. Adult patients who were initially seen in the established adult neuro-oncology clinic were transferred to the AYA clinic in the presence of certain tumor markers or cancer predisposition syndromes seen in the final pathology or pathology review and/or as discussed in the regular multidisciplinary rounds. Referrals also came from regional neurosurgeons and radiation oncologists, as well as external medical centers. There is an integrated network within the clinic connecting the patients with an adult neurologist specialized in neuro-oncology, neurosurgeons, radiation oncologists, specialists in different genetic syndromes, as well as support services including a neuropsychologist, social worker, clinical dietician, and rehabilitation specialist. Patients in need of fertility counseling are referred to the hospital-wide AYA cancer program.^20^ Practitioners from this program give fertility counseling to patients and facilitate referrals to specialized fertility clinics when appropriate.^21^ The clinic works closely with neuroradiologists who interpret different brain and spine imaging and perform additional tests such as MR perfusion studies when necessary as well as neuropathologists who ensure that relevant molecular testing is performed when applicable to guide treatment decisions, including next-generation sequencing or methylation array profiling. Patients, specifically those with progressive disease, are referred to neurosurgeons and radiation oncologists. Cases are discussed as appropriate with neurosurgeons, radiation oncologists, neuroradiologists, neuropathologists, and allied health teams through weekly clinic rounds and multidisciplinary tumor boards. Clinical trials are offered to patients when available. There are devoted clinical trial nurses who help in the process of screening and monitoring clinical trial patients (Figure 1).

### Demographic profile

A total of 100 patients were seen in the AYA neuro-oncology clinic from December 2022 to June 2024 and all are included in the study (54% male; median age 24 years, range 17–79; Table 1). Ninety patients (90%) were within the globally accepted age range of AYA (15–39 years).^1^ For patients aged 40 and older, referral to the AYA clinic was based on having rare tumors more commonly observed in younger patients and the need for treatments more commonly administered in the pediatric setting. Specifically, they were referred for these reasons: 3 cases for diagnostic workup with liquid CSF biopsy, 2 cases of glioma with mismatch repair deficiency, 2 cases of ependymoma, and 1 case each of glioma with H3K27M mutation, craniopharyngioma for intracystic interferon therapy, and low-grade glioma (LGG) with BRAF mutation. Sources of referrals were from the following: 18% were transitioning to AYA care from an affiliate pediatric hospital, 25% were newly diagnosed and referred directly to the clinic, 41% were transfers of care from other specialists such as neurosurgeons and radiation oncologists, and 16% were transfers of care from adult neuro-oncology in the same hospital. Of note, 5 out of 16 (31%) in the last group were newly diagnosed patients who were referred initially to adult neuro-oncology and transferred immediately to the AYA clinic after initial assessment.

**Table 1.** Demographic Profile of Patients

Demographics	Median [range], *n* (%)
*Age, years*	24 [17–79]
15–39 years	90 (90%)
40 years and older	10 (10%)
*Sex*	
Male	54 (54%)
Female	46 (46%)

### Diagnosis, Pathology, and Molecular Findings

Seventy-six patients had diagnoses confirmed by biopsy, including pediatric transition patients who had surgery before referral to adult care. Among these patients, the most common diagnoses were LGG (59%), high-grade glioma (HGG; 13%), and medulloblastoma (8%). Pilocytic astrocytoma and glioneuronal/neuronal tumors were the most common LGGs (Table 2). Eight patients had no tissue biopsy but were presumed to have LGG from the radiographic findings.

**Table 2.** Tumor Characteristics

Histopathology	*n* (%)
Low-grade glioma Pilocytic astrocytoma Glioneuronal/neuronal tumor Pleomorphic xanthoastrocytoma Diffuse astrocytoma Subependymal giant cell astrocytoma Oligodendroglioma Unspecified	45 (59.21%) 18 (40%) 10 (22.22%) 3 (6.67%) 2 (4.44%) 1 (2.22%) 1 (2.22%) 10 (22.22%)
High-grade glioma Glioblastoma Diffuse midline glioma Diffuse high-grade glioma	10 (13.16%) 6 (60%) 2 (20%) 2 (20%)
Embryonal tumors Medulloblastoma Pineoblastoma	9 (11.84) 6 (66.67%) 3 (33.33%)
Germ cell tumor	5 (6.58%)
Ependymoma	3 (3.95%)
Craniopharyngioma	2 (2.63%)
Atypical teratoid rhabdoid tumor	1 (1.32%)
Diffuse leptomeningeal glioneuronal tumor	1 (1.32%)
**Molecular alteration**	***n* (%)**
FGFR TKD duplication	1 (2.04%)
FGFR mutation	9 (18.37%)
FGFR fusion	3 (6.12%)
BRAF mutation	11 (22.45%)
BRAF fusion	11 (22.45%)
H3K27M mutation	2 (4.08%)
PTPN11 mutation	3 (6.12%)
PTEN mutation	1 (2.04%)
MMRD	6 (12.24%)
RAF1 fusion	1 (2.04%)
NKTR2 fusion	1 (2.04%)
PIK3CA mutation	3 (6.12%)
KRAS mutation	1 (2.04%)
NF1	3 (6.12%)
TSC	1 (2.04%)
IDH mutation	2 (4.08%)
**Location on imaging review**	***n* (%)**
Unifocal Lobar Suprasellar Thalamic, epithalamic (pineal) Brainstem Cerebellar Intraventricular Spinal cord	78 (78%) 28 (35.9%) 15 (19.23%) 9 (11.54%) 8 (10.26%) 11 (14.1%) 4 (5.13%) 3 (3.85%)
Multifocal/ Disseminated Lobar Suprasellar Subcortical Thalamic, epithalamic (pineal) Brainstem Cerebellar Intraventricular Spinal cord Leptomeningeal	22 (22%) 3 (13.64%) 8 (36.36%) 2 (9.09%) 11 (50%) 2 (9.09%) 4 (18.18%) 6 (27.27%) 4 (18.18%) 3 (13.64%)

Molecular alterations were found in 49 patients (89%) with glioma or glioneuronal tumors. The most common variants were BRAF mutation (*n* = 11) and BRAF fusion (*n* = 11). FGFR mutation was documented in 9 patients (Table 2). Of biopsy-confirmed LGG (*n* = 45), 40 had molecular alterations. The remaining 5 were confirmed LGG on histopathology (pilocytic astrocytoma-2, dysplastic cerebellar gangliocytoma-1, unspecified-2) with no identified molecular alterations on testing.

Among the 6 patients diagnosed with medulloblastoma, 4 patients were classified as sonic hedgehog (SHH)-activated tumors, and 2 patients were classified as group 4. Among the 3 patients with pineoblastoma, 2 patients were classified as group 2 and 1 patient was classified into group 3.^22^ Among the 3 patients diagnosed with ependymoma, 2 were classified as ZFTA-fusion positive and 1 was classified as posterior fossa ependymoma group B (PFB). Only 1 patient with craniopharyngioma had immunohistochemistry staining revealing beta-catenin positive and BRAF V600E negative.

### Imaging Review and Tumor Location

Most patients (78%) had unifocal disease with the remainder having multifocal or disseminated disease. Among those with unifocal disease, the majority (36%) had lobar lesions, followed by suprasellar (19%) and cerebellar lesions (14%). The thalamic and pineal regions (50%), suprasellar (36%), and intraventricular region (27%) were mainly involved among patients with multifocal or disseminated disease (Table 2). The most common primary location of the tumor was the pineal gland (27%) among those with multifocal disease.

### Cancer Predisposition

Twenty-three patients (23%) had germline cancer predisposition syndromes and were referred for genetic counseling. The most common conditions were neurofibromatosis type 1 (NF1) and glioma with mismatch repair deficiency (Table 3). The recognition of cancer predisposition syndrome is crucial as it affects treatment options. Specifically, glioma patients with mismatch repair deficiency are offered immune checkpoint inhibitors. In the same way, patients with tuberous sclerosis complex and NF1 are referred to the comprehensive Tuberous Sclerosis and Neurofibromatosis clinics, respectively.

**Table 3.** Cancer Predisposition

Cancer predisposition	*n* (%)
ATRT SMARCB1 germline mutation	1 (4.35%)
Pineoblastoma Group 2 with DICER 1 germline mutation	1 (4.35%)
Tuberous sclerosis complex	4 (17.39%)
Neurofibromatosis 1	9 (39.13%)
Possible Cowden’s syndrome	1 (4.35%)
Lynch syndrome	6 (26.09%)
Li-Fraumeni syndrome	1 (4.35%)

### Treatment

Seventy-seven patients underwent a minimum of one surgical procedure. Biopsy was the most frequent (31%), followed by subtotal resection (29%). Fifteen patients underwent more than one surgical intervention. Thirty-one patients underwent radiotherapy with the most common modality being conventional linear accelerator (LINAC) radiation therapy with photons. Focal radiation therapy was the most common intervention provided by volume (67%). Table 4 summarizes the surgical and radiation oncology treatment profiles of all patients.

**Table 4.** Interventions

Interventions	*n* (%)
Surgery Biopsy Subtotal resection Gross total resection Combined Others	77 (77%) 24 (31.17%) 22 (28.57%) 12 (15.58%) 15 (19.48%) 4 (5.19%)
Radiation therapy By Modality LINAC only Proton only Both LINAC and proton ◦ LINAC, Conventional ◦ LINAC, SRS By Volume • Focal • CSI • WVRT • Combined	31 (31%) 27 (87.1%) 2 (6.45%) 2 (6.45%) 28 1 21 (67.74%) 6 (19.35%) 1 (3.22%) 3 (9.68%)
Chemotherapy Single line only Second line or more	43 (43%) 29 (67.44%) 14 (32.56%)
Targeted treatment Trametinib monotherapy Binimetinib monotherapy Pemigatinib monotherapy Dabrafenib + Trametinib Selumetinib monotherapy Dabrafenib monotherapy Everolimus	21 (21%) 12 (57.14%) 1 (4.76%) 2 (9.52%) 3 (14.29%) 2 (9.52%) 1 (4.76%) 2 (9.52%)
Other treatments Pembrolizumab Bevacizumab Nivolumab Nivolumab + Ipilimumab Intracystic Bleomycin Intracystic Interferon Other immunotherapy	13 (13%) 1 (7.69%) 5 (38.46%) 3 (23.08%) 1 (7.69%) 1 (7.69%) 1 (7.69%) 1 (7.69%)

CSI, craniospinal irradiation; WVRT, whole-ventricular radiotherapy.

Overall, forty-three patients were given chemotherapy, including patients who received therapy prior to referral and patients who started therapy after referral to the clinic (Table 4). Among these patients, 20 were diagnosed with LGG, 8 patients with HGG, 6 patients with medulloblastoma, 5 patients with germ cell tumor, 3 patients with pineoblastoma, and 1 patient with ATRT. Most of the patients (67%) received only a first-line chemotherapy regimen. Vinblastine monotherapy was the most common chemotherapy received by patients diagnosed with LGG and this was administered while under pediatric care. Temozolomide was given to most patients with HGG. Twenty-one patients received targeted treatment with trametinib monotherapy being the most common medication given (57%). This is followed by a combination of dabrafenib and trametinib (14%). Targeted therapy was offered to patients at diagnosis in 4 patients (19%) and at disease progression in 17 patients (81%). Eleven patients received other therapies which include bevacizumab, immunotherapy (pembrolizumab, nivolumab), or intracystic bleomycin and interferon therapies.

### Therapies Started in the AYA Neuro-oncology Clinic

Twenty-nine patients (29%) were initiated on systemic therapy in the AYA clinic. Of the newly diagnosed patients, 38% were prescribed treatment. The remainder were either transferred from the adult neuro-oncology clinic (17.24%), from the affiliate pediatric hospital (10.34%), or other clinics (34.48%) for ongoing observation. Table 5 summarizes the treatments prescribed to these patients. Of note, 1 patient diagnosed with medulloblastoma declined adjuvant chemotherapy. Two patients with HGG (glioblastoma from Lynch syndrome) were planned to receive immunotherapy but management has not commenced at the time of writing. Lastly, 4 patients with HGG did not receive treatment because they were either stable on surveillance (25%), had poor functional status precluding chemotherapy (25%), or were transferred back to an established adult neuro-oncology clinic for treatment (50%).

**Table 5.** Medical Therapy Prescribed in the Adolescents and Young Adults Neuro-oncology Clinic

Number of patients (% of all patients)	Diagnosis	Therapy
15 (23.81%)	Glioma	11 patients given targeted therapy 2 patients given immunotherapy 2 patients enrolled in trial
5 (100%)	Germ cell tumor	All patients given ACNS1123 treatment protocol^23,24^
5 (83.33%)	Medulloblastoma	1 patient given etoposide at recurrence 4 patients given ACNS0332 treatment protocol^25^
3 (100%)	Pineoblastoma	All patients given ACNS0332 treatment protocol^25^
1 (100%)	Craniopharyngioma	1 patient given intracystic interferon

### Clinical Trial Enrollment

Three patients were eligible for clinical trials and all were enrolled. They were diagnosed with glioblastoma, diffuse midline glioma, and LGG with BRAF fusion. The patient with glioblastoma was transferred back to the established adult neuro-oncology service after enrollment and was not actively managed by the AYA clinic. Therefore, only 2 out of 29 patients (7%) who were actively receiving treatment in the AYA clinic were eligible for a clinical trial.

### Referrals to Services

Seventeen patients were referred to neuropsychology for a comprehensive neurocognitive assessment. Most of these patients (58.82%) were diagnosed with LGG and about a third (29.41%) of the patients received intensive chemotherapy. Recommendations from this testing were used to guide patients as they return to school or work and to tailor strategies to manage cognitive symptoms in daily living. Eight patients were referred to a clinical dietician. Seven of these patients were actively treated in the AYA clinic with intensive chemotherapy and deemed high risk for malnutrition. This comprises around 41% of patients who were actively treated in the clinic with more intensive therapy (diagnosed with HGG, embryonal tumors, and germ cell tumors). Nine patients were referred to the fertility clinic for a more thorough discussion on fertility assessment and fertility preservation. Seven of these patients received intensive chemotherapy in the AYA clinic which could potentially have consequences on fertility status. Four other patients who received active treatment in the clinic were also referred to the hospital-wide AYA service for initial discussions on general fertility counseling while on treatment but were not necessarily referred to fertility specialists. Fourteen patients were referred to rehabilitation or physiotherapy services. Thirty-one patients were referred to psychosocial oncology which involved referral to a social worker or psychiatrist. The social worker helped by providing psychosocial support as well as navigating school and work transitions.^26^ The role of the psychiatrist is centered around the diagnosis of psychiatric conditions and medical management of psychiatric symptoms.

### Challenges

There are several challenges in the conduct of the multidisciplinary neuro-oncology AYA clinic. Clinical trial restrictions, especially in terms of age, limit the enrollment of AYA patients in studies. There were also some difficulties encountered in accessing new targeted medications despite evidence for use in certain tumor types leading to delays in initiation of treatment for some patients. AYA are also less likely to have private drug insurance than younger children or older adults, limiting their access to some medications, such as bevacizumab, that are not readily covered by public insurance for certain indications in Ontario (such as tumor inflammatory-associated neurotoxicity during immunotherapy^27^) often limiting the number of infusions.^28^ Despite the available support services in the clinic, referral rates to these services are low. Whether this is because AYAs do not require these services, are unaware of these services, or are not being systematically screened/referred remains to be explored. Lastly, peer support groups specifically dedicated to AYA patients with CNS tumors seem to be lacking.

## Discussion

There is very limited literature addressing the different aspects of care among AYA neuro-oncology patients despite the significant interest in the care of AYA cancers globally. The formation of dedicated AYA neuro-oncology clinics in cancer centers fostering multidisciplinary care has been recommended to improve the quality of care^6,7,19^; however, there has been no report documenting the conduct of such a clinic or the feasibility of such a framework to date. The multidisciplinary AYA neuro-oncology clinic at the Princess Margaret Cancer Center was established to provide specialized and holistic care to AYAs newly diagnosed with “pediatric-type” tumors and to AYAs with these diagnoses who transition from pediatric to adult clinic settings. Since its inception, the clinic has provided services to 100 patients, either directly referred to the clinic or transferred from other centers. In the coming years, the influx of more referrals is expected from the affiliate pediatric and adult neuro-oncology clinics where 114 (Male: 61; Female: 53) and 481 (Male: 276; Female: 205) new patients were seen, respectively during the same 18-month period. The adult neuro-oncology clinic has been established for about 30 years and most pediatric patients transitioned here prior to the opening of the AYA clinic. As much as 26% of all patients seen in the adult clinic during the same 18-month period were 39 years or younger. Of the AYA patients seen, 66% were follow-up cases from the prior years. Interestingly, a few patients older than 40 years with confirmed or suspected pediatric-type tumors or tumors with alterations more commonly seen among the pediatric age group were also referred to the AYA clinic. This highlights that the combined expertise of pediatric and adult neuro-oncologists enhances care for patients older than the globally accepted definition of AYA (15–39 years old). Furthermore, this emphasizes that care for brain tumor patients should not be compartmentalized strictly by age. As tumor types and molecular features may overlap along the spectrum of age groups, inputs from AYA specialists may play a vital role in management, especially among rare tumors.

In the current era of novel targeted therapy, the identification of oncogenic drivers becomes important in personalizing treatments.^6,8,29^ The AYA clinic offers comprehensive tumor molecular characterization for patients, along with the opportunity for multidisciplinary case review which plays a crucial role in identifying these mutations and offering targeted therapy when appropriate. Molecular alterations were found in 86.6% of biopsy-confirmed LGGs seen in the clinic, with the most common alteration being BRAF fusion and BRAF V600E mutation. The use of MEK inhibitors, alone or in combination with BRAF inhibitors, has been used for these MAPK pathway-altered tumors.^30–32^ In the clinic, trametinib monotherapy was primarily given followed by the combination of trametinib and dabrafenib. FGFR alteration was also documented in 13 patients. Pemigatinib is a selective FGFR 1–3 inhibitor which was found to have antitumor activity on progressive tumors including CNS malignancy.^33^ In the setting of progressive disease and the presence of targetable mutation, pemigatinib has been given to selected patients in the AYA clinic, provided by a compassionate use program. The result of FIGHT-209 trial (NCT05267106),^34^ a phase 2 study on the efficacy and safety of pemigatinib among patients with progressive CNS tumors harboring FGFR alterations is eagerly anticipated. Another phase 2 study (NCT06653777)^35^ on the efficacy of pemigatinib among solid tumors with FGFR alterations is expected to start recruitment soon. The results of these trials will hopefully provide more insight into the utility of this drug among glioma patients.

A recent review on the clinical trial accrual rate of AYAs in Canada showed that from 2004 to 2022, of the Canadian Cancer Trials Group studies available to AYA neuro-oncology patients, participation was minimal at 2% of 115 enrolled patients.^36^ Age restrictions in clinical trials which often exclude AYAs remain a significant hindrance in maximizing clinical trial enrollment among patients. Further, AYAs may have “pediatric-type” tumors which are not readily addressed by trials designed for the adult patient population.^14^ Only 7% of patients who were actively treated in our AYA clinic were eligible and were enrolled in a clinical trial. This highlights the need for more AYA inclusivity in designing neuro-oncology clinical trial protocols with the aim of establishing the best practice for this unique patient subgroup.^6^

Integrating psychosocial support, rehabilitation, nutrition, fertility counseling, and neuropsychological assessment are central to the multidisciplinary model of care of this clinic. There are several themes unique to the psychosocial needs of AYAs namely feelings of isolation and uncertainty about the future, the need for more information and autonomy when discussing with the medical team, cultivating a clinic setup more inclusive of AYA patients, and unique needs of AYA patients who identify themselves as LGBTQ+.^37^ These themes underscore the need for personalized care from the physician and allied health teams. In the AYA clinic, patients who express the need for more psychosocial support are referred to the clinic social worker who is part of the psychosocial oncology service. Depending on their needs, patients may be connected to community resources or referred to a psychiatrist. While the Brain Tumor Foundation of Canada offers an online support group exclusive for young adult patients,^38^ there is a lack of organized peer support group specifically among AYA patients with brain tumors in the setting of the AYA neuro-oncology clinic and this is an important endeavor to work on in the future. This has been particularly evident in patients transitioning from pediatric care into AYA care, as their prognosis varies dramatically from many of their peers with newly diagnosed glioma who may face a shortened life span, coupled with the fact that these are patients who have potentially already undergone many years of therapy at the time of transition.

A study on 575 Canadian AYA patients including those diagnosed with CNS malignancy showed that fatigue was the most reported physical concern post-treatment.^39^ This makes access to physical therapy assessments and interventions as well as referrals to local rehabilitation teams significant services available to AYA patients in the clinic. AYAs are also at risk of nutritional problems specifically malnutrition and obesity during and after treatment, respectively.^40^ Referral to the clinical dietician becomes an important resource not just to mitigate the effects of nutrition changes caused by treatment but also because body image, specifically physical body appearance, can be a particularly important part of well-being in this age group. A meta-analysis on the fertility of CNS cancer patients revealed that the pooled prevalence of gonadal toxicity was 20%, justifying the need to discuss fertility preservation among this patient population.^41^ In the AYA clinic, access to fertility specialists for discussion on the possible effects of therapy on reproductive health as well as possible methods for fertility preservation are made available to patients. Changes in memory and concentration were reported by almost half of the Canadian AYA cancer survivors in a nationwide survey.^39^ The conduct of serial comprehensive neuropsychological evaluations is an integral part of the management of brain tumor patients and is a resource that should be offered to patients on active therapy and surveillance given the neurocognitive implications of diagnosis and treatment.^42^ These neurocognitive changes can profoundly impact patient functioning and autonomy, often leading to reduced quality of life. Neuropsychological assessment, readily made available to AYA patients in the clinic, can evaluate and characterize these neurocognitive sequelae and offer interventions and recommendations to improve functioning and quality of life.^43^ Finally, while allied health teams are available in the multidisciplinary AYA clinic, referrals made have been limited and may be further maximized. For instance, most of the referrals sent to these services were done in the context of starting intensive chemotherapy. However, AYAs are all vulnerable to varying stressors regardless of diagnosis and extent of therapy and concerns on fertility,^39^ nutrition,^40^ cognition,^42^ physical activity level,^39^ and psychosocial needs^37^ may extend through survivorship.

## Limitations

The study relied on the retrospective review of medical records available in the hospital’s electronic database. Consequently, there may be some data missing from the available records which may include some laboratory findings done in outside institutions which are not accessible and not linked to the patient’s available records. A formal mixed methods needs assessment including perspectives from AYA health care providers and AYA patients would be helpful in further documenting gaps in AYA neuro-oncology care. Finally, a study analyzing the outcomes of patients treated in the multidisciplinary AYA clinic in comparison with patients treated in adult centers alone is necessary to fully prove the advantage of this new approach.

## Conclusion

This is the first study to document the clinic experience and patient profile in a multidisciplinary AYA neuro-oncology clinic setting. The establishment of this multidisciplinary team with both pediatric and adult neuro-oncology specialists, together with radiation oncologists, neurosurgeons, neuroradiologists, neuropathologists, and allied health teams, has been a pioneering endeavor to address the fragmented care of AYA patients as described in the literature. At the time of writing, the clinic has been established for approximately 18 months and has served 100 patients. More patients are expected to be referred to the clinic in the coming years as the unique needs of AYA neuro-oncology patients are recognized better and the advantages of multidisciplinary care become evident. Clinical trials need to be more inclusive of AYA patients. This will lead to higher enrollment rates among AYA and ultimately, improved treatments for this vulnerable group. Continuing to increase awareness of the supportive care needs of AYA and developing programs to meet those needs serve as further goals in the coming years.

## Data Availability

Data will be made available upon request by emailing the corresponding author.
